# Genomics advances the study of inbreeding depression in the wild

**DOI:** 10.1111/eva.12414

**Published:** 2016-10-23

**Authors:** Marty Kardos, Helen R. Taylor, Hans Ellegren, Gordon Luikart, Fred W. Allendorf

**Affiliations:** ^1^ Department of Evolutionary BiologyEvolutionary Biology CentreUppsala UniversityUppsalaSweden; ^2^ Department of AnatomyUniversity of OtagoDunedinNew Zealand; ^3^ Division of Biological SciencesUniversity of MontanaMissoulaMTUSA; ^4^ Flathead Lake Biological StationDivision of Biological SciencesUniversity of MontanaPolsonMTUSA

**Keywords:** conservation genetics, fitness, identity by descent, pedigree analysis, whole‐genome resequencing

## Abstract

Inbreeding depression (reduced fitness of individuals with related parents) has long been a major focus of ecology, evolution, and conservation biology. Despite decades of research, we still have a limited understanding of the strength, underlying genetic mechanisms, and demographic consequences of inbreeding depression in the wild. Studying inbreeding depression in natural populations has been hampered by the inability to precisely measure individual inbreeding. Fortunately, the rapidly increasing availability of high‐throughput sequencing data means it is now feasible to measure the inbreeding of any individual with high precision. Here, we review how genomic data are advancing our understanding of inbreeding depression in the wild. Recent results show that individual inbreeding and inbreeding depression can be measured more precisely with genomic data than via traditional pedigree analysis. Additionally, the availability of genomic data has made it possible to pinpoint loci with large effects contributing to inbreeding depression in wild populations, although this will continue to be a challenging task in many study systems due to low statistical power. Now that reliably measuring individual inbreeding is no longer a limitation, a major focus of future studies should be to more accurately quantify effects of inbreeding depression on population growth and viability.

## 1 Introduction

Inbreeding (mating between relatives) generally causes offspring to have reduced fitness (Charlesworth & Willis, 2009; P. W. Hedrick & A. García‐Dorado, in press; Keller & Waller, 2002). This phenomenon, known as inbreeding depression, can be caused by increased homozygosity at loci with deleterious recessive alleles, or decreased heterozygosity at loci displaying heterozygous advantage (Charlesworth & Willis, 2009). Small populations, where most or all mates are relatively closely related, are particularly vulnerable to inbreeding and inbreeding depression (Keller & Waller, 2002). The total effects of inbreeding depression on individuals in small populations can accumulate to reduce the population growth rate and increase the probability of extinction (Frankham, 1995, 2005; Hedrick & Kalinowski, 2000; Keller & Waller, 2002; Mills & Smouse, 1994; O'Grady et al., 2006; Saccheri et al., 1998; Soulé & Mills, 1998; Westemeier et al., 1998). Despite being of interest since Darwin (1896), inbreeding depression remains a crucial area of research in conservation biology, ecology, and evolutionary biology. As global change and habitat destruction and fragmentation rapidly progress, many natural populations will become smaller and more isolated (Haddad et al., 2015) and consequently more affected by inbreeding depression.

Surprisingly little is known about the severity of inbreeding depression in the wild, in part because it is very difficult to measure individual inbreeding in natural populations. The genetic architecture of inbreeding depression and the effects of inbreeding depression on population growth are also still not well understood (Charlesworth & Willis, 2009; Hedrick & Kalinowski, 2000; Keller & Waller, 2002). For example, how many genes generally contribute to inbreeding depression, and how frequently is inbreeding depression caused by alleles with large effects? How much of inbreeding depression is due to homozygosity at loci with deleterious recessive alleles versus homozygosity at loci displaying heterozygous advantage? A crucial area of research in conservation biology is to determine how frequently the fitness components depressed by inbreeding strongly affect the population growth rate. A comprehensive understanding of the causes and consequences of inbreeding depression will require an understanding of the underlying genetic basis and its effects on population growth and viability.

The availability of large‐scale molecular genetic data is dramatically changing our ability to measure individual inbreeding and inbreeding depression in the wild. Pedigree‐based analyses have traditionally been the cornerstone of studies on individual inbreeding (Pemberton, 2004, 2008; Slate et al., 2004). The pedigree inbreeding coefficient (*F*_P_) predicts the probability of a locus being “identical‐by‐descent” (IBD) based on a known pedigree where the founders are assumed to be unrelated and noninbred (Keller & Waller, 2002; Malécot, 1970; Wright, 1922). A locus is said to be IBD if the two homologous gene copies within an individual arise from a single copy in a common ancestor of the parents. *F*_P_ cannot perfectly predict the actual proportion of the genome that is IBD (*F*), because of linkage and limited pedigree depth (Forstmeier, Schielzeth, Mueller, Ellegren, & Kempenaers, 2012; Franklin, 1977; Hill & Weir, 2011; Stam, 1980; Box 1). Additionally, pedigrees are difficult to obtain for natural populations because they require reliable parentage information across several generations (Pemberton, 2008). Fortunately, it is now possible to type thousands of loci (Andrews, Good, Miller, Luikart, & Hohenlohe, 2016) or sequence the genomes of many individuals in any natural population (e.g. Ellegren, 2014; Kardos, Husby, McFarlane, Qvarnström, & Ellegren, 2015; Lamichhaney et al., 2015). This huge amount of molecular genetic data can be used to precisely measure individual inbreeding via analysis of genetic variation across individual genomes (Bérénos, Ellis, Pilkington, & Pemberton, 2016; Hoffman et al., 2014; Huisman, Kruuk, Ellis, Clutton‐Brock, & Pemberton, 2016; Kardos, Luikart, & Allendorf, 2015; Keller, Visscher, & Goddard, 2011; Kirin et al., 2010; Knief et al., 2015; McQuillan et al., 2008, 2012; Pemberton et al., 2012), and to study inbreeding depression without needing to conduct parentage analysis over many generations.

Box 1 **Genomic signatures of individual inbreeding**

**Figure 1:**
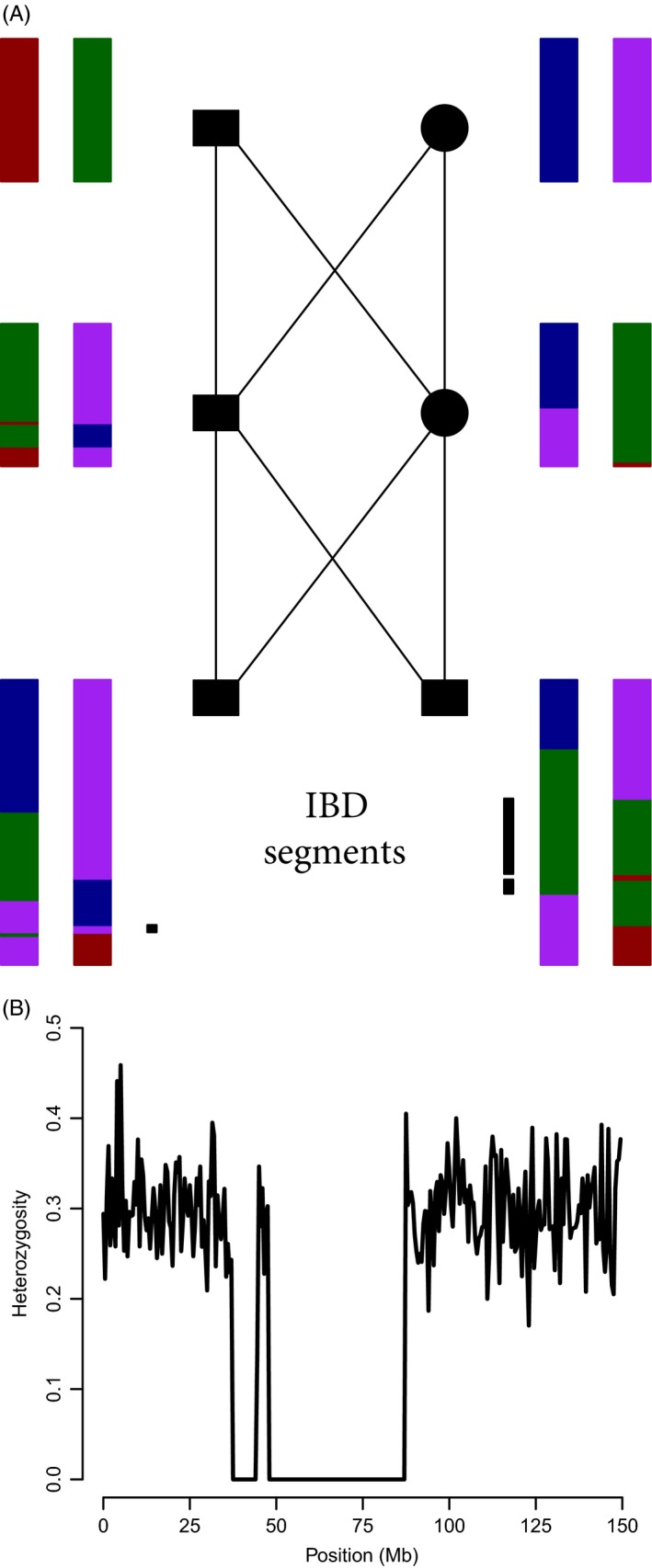
(A) The simulated inheritance of chromosomes of two brothers (bottom squares) whose parents are full siblings. The grandparents each have two unique copies of a single 150‐Mb, 180‐cM chromosome (represented by different colors). The locations of recombination events are represented by the boundaries between different colors in the chromosomes of the offspring. The inbred brother on the left has one IBD chromosome segment (generating one long ROH), and the brother on the right has two (mapped with black bars). (B) The distribution of heterozygosity across the chromosome of the inbred brother on the right in (A). Heterozygosity (*y*‐axis) is the proportion of heterozygous SNPs in nonoverlapping 500‐kb windows. IBD segments are identified as regions with no heterozygous SNPs. Simulation details are available in the Supporting Information

1 Inbreeding causes offspring to have identical‐by‐descent (IBD) chromosome segments, which are characterized by long “runs of homozygosity” (ROH) at mapped SNPs (Fig. 1). IBD chromosome segments occur where the parents transmit identical copies of a chromosome segment that both arise from a single copy in a common ancestor. Recombination events and Mendelian sampling of chromosome copies along the genealogy separating the inbred individual from the common ancestor(s) of the parents determine the boundaries of IBD segments (Fig. 1). Linkage increases the variation in *F* among individuals with identical pedigrees (Franklin, 1977; Hill & Weir, 2011; Stam, 1980). The variance in *F* among individuals with identical pedigrees is highest in organisms with few chromosomes and low recombination rate (Franklin, 1977; Hill & Weir, 2011; Stam, 1980). This is because having fewer chromosomes results in a larger fraction of the genome exhibiting nonindependent segregation. The same is true for recombination rate—less recombination means that loci on the same chromosome are more likely to segregate together during meiosis.

**Figure 2:**
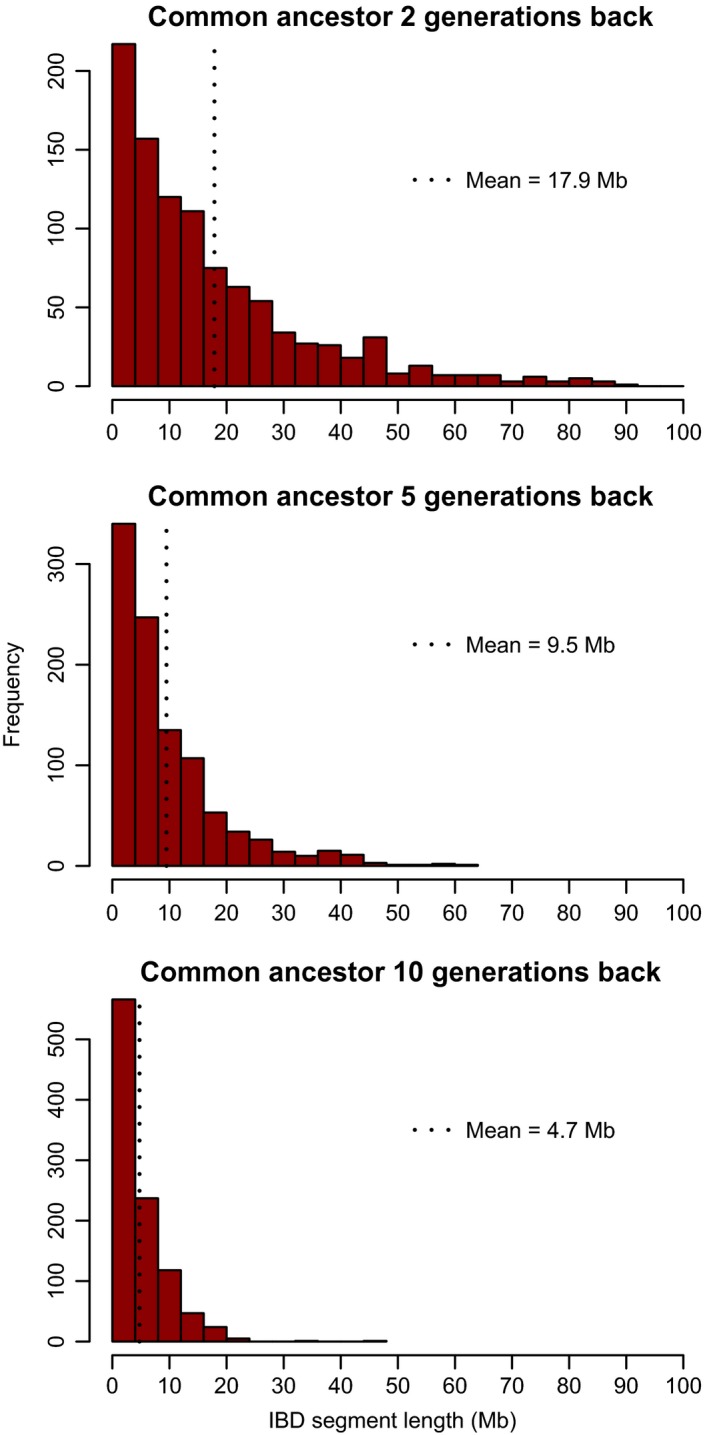
The distribution of the lengths of IBD segments arising from a common ancestor 2, 5, or 10 generations back. The simulated genomes included ten 150‐Mb, 180‐cM chromosomes. Details of the simulations are given in Supporting Information

The length of IBD chromosome segments is strongly influenced by the number of generations separating the inbred individual from the common parental ancestor(s). Inbreeding due to recent ancestors usually generates quite long IBD chromosome segments, whereas IBD segments deriving from more distant ancestors tend to be shorter on average because of a greater number of meioses, and recombination events, separating the inbred individual from the parental ancestor (Fig. 2). The map lengths of IBD segments arising from ancestors *g* generations ago are exponentially distributed with mean (100/2 *g*) cM (Thompson, 2013), but there is a high variance around this expected value due to the stochastic nature of recombination and Mendelian segregation (Figs 1 and 2). *g* is equivalent to the “time to the most recent common ancestor” (TMRCA) of the two homologous copies of DNA within an IBD chromosome segment.

Given the rapid growth of genomic resources available for studies of nonmodel organisms, now is an excellent time to appraise the future prospects for molecular genetic‐ and pedigree‐based studies of inbreeding and inbreeding depression in the wild. Here, we review recent developments in (i) how the availability of large‐scale genomic data has led to a change in the way individual inbreeding is defined, (ii) the performance of molecular marker‐based measures of *F* relative to *F*_P_, and (iii) mapping loci responsible for inbreeding depression and how such approaches may be applied in natural populations. Throughout, we highlight the opportunities and challenges that genomics provides for the study of inbreeding and inbreeding depression in the wild.

## 2 Defining Individual Inbreeding (*F*)

An individual is “inbred” if its parents share a common ancestor(s) (Keller & Waller, 2002). The inbreeding of an individual has traditionally been defined parametrically by the pedigree inbreeding coefficient *F*_P_. Note that there are other definitions of inbreeding, and there has been considerable and persistent confusion in the interpretation of inbreeding coefficients (Jacquard, 1975; Keller & Waller, 2002; Templeton & Read, 1994). In Box 2, we describe other forms of inbreeding and how they are related to individual inbreeding.

Box 2 **Alternative definitions of inbreeding and their relationship to individual inbreeding**

1 There are multiple definitions of the word inbreeding (Jacquard, 1975; Keller & Waller, 2002), which has caused considerable confusion in the interpretation of the various inbreeding coefficients (Templeton & Read, 1994). We believe it is important here to clearly distinguish individual inbreeding from other major types of inbreeding.

**Inbreeding as Nonrandom Mating Within a Population**

Inbreeding can be defined as mating between individuals who are more closely related than the average randomly selected pair of individuals within a population. This form of inbreeding occurs, for example, in populations where self‐fertilization occurs more often than expected by chance. Inbreeding as nonrandom mating is typically measured with the inbreeding coefficient *F*_IS_. *F*_IS_ ranges from −1 to 1, with positive values indicating that mates are more closely related on average than expected, and a deficit of heterozygotes relative to Hardy–Weinberg proportions. Negative *F*_IS_ indicates that mates are on average less closely related than expected by chance, resulting in an excess of heterozygotes relative to Hardy–Weinberg proportions.

*F*_IS_ is frequently estimated in conservation genetic studies of small populations, but is seldom clearly differentiated from an estimate of the extent of individual inbreeding (e.g. Hudson, Vonlanthen, & Seehausen, 2014; Paiva, da Silva Mariante, & Blackburn, 2011; Potter et al., 2012; Vonholdt et al., 2008). The distinction between individual inbreeding and inbreeding as nonrandom mating is crucial. For example, a high mean *F* is expected in random mating populations that have been small for many generations because all individuals will be closely related, but the expected *F*_IS_ is zero because the current population is taken as the base population against which mean individual heterozygosity is compared (Felsenstein, 2015, p. 266). In fact, *F*_IS_ is expected to be negative in very small populations because of allele frequency differences between males and females (Balloux, 2004; Robertson, 1965). Large positive values of *F*_IS_ occur when individuals are sampled from multiple, large, genetically differentiated populations (i.e. the Wahlund effect, Felsenstein, 2015, p. 165). Thus, *F*_IS_ should not be considered as a measure of the extent of individual inbreeding in a population.

**Inbreeding as Population Subdivision**

Population subdivision causes mates to be more closely related than when mating is random among all individuals (i.e. in a panmictic population). Inbreeding arising from within‐population genetic drift and differentiation among subpopulations in the classic “infinite island” model of gene flow is quantified by Wright's *F*_ST_ (Felsenstein, 2015, p. 266). The base population for *F*_ST_ is defined by the allele frequencies averaged across all subpopulations. Mean *F* will equal *F*_ST_ in the infinite island model, where we assume equal population sizes and random mating within populations.

Inferring the extent of individual inbreeding from *F*_ST_ is rarely useful in practice. First, high *F*_ST_ can result from rapid genetic differentiation among populations with very small *N*_e_ (with high mean individual *F* due to recent ancestors), or as a result of genetic drift over very long time spans among genetically diverse populations with large *N*_e_ and very little individual inbreeding arising from recent ancestors. Additionally, the assumptions of the infinite island model (equal population sizes and random mating within populations) are usually violated in real populations.

Proper interpretation of empirical estimates of *F*_IS_ and *F*_ST_ requires careful definition of the base population. Estimates of individual genetic variation based on genomewide heterozygosity or ROH are much more informative of the extent of individual inbreeding than the population *F*‐statistics *F*_IS_ and *F*_ST_ (see 4 in the main text).

An obvious problem with defining individual inbreeding according to a particular pedigree is that loci can be IBD due to more distant ancestors than those included in the pedigree. *F*_P_ will therefore generally underestimate the actual proportion of the genome that is IBD (*F*). An additional problem with defining individual inbreeding relative to a pedigree is that *F* can vary substantially among individuals with identical pedigrees because of the effects of linkage (Fisher, 1965, p. 97; Forstmeier et al., 2012; Franklin, 1977; Hill & Weir, 2011; Kardos, Luikart, et al., 2015; Stam, 1980; see Box 1). Thus, even a pedigree that includes all common ancestors of parents cannot perfectly predict *F*. Recently, increased acknowledgement of these problems with the pedigree‐based parametric definition of individual inbreeding has led to the recognition that *F* (and not *F*_P_) is the most appropriate parameter for studies of inbreeding depression (Forstmeier et al., 2012; Hill & Weir, 2011; Kardos, Luikart, et al., 2015; Wang, 2016).

The concept of *F* as a parametric definition of individual inbreeding is somewhat complicated by the fact that all pairs of homologous gene copies derive from a single ancestral copy at some point in the past. There is a continuum of the “time to the most recent common ancestor” (TMRCA) for homologous loci across an individual's genome (Speed & Balding, 2015). Chromosome segments with a TMRCA of only a few generations tend to be very long (Box 1) and to be devoid of heterozygous positions. Chromosome segments with very large TMRCA (e.g. hundreds to thousands of generations) tend to be very short and are more likely to contain heterozygous positions arising from mutations along one or the other of the parental lineages reaching back to the common ancestral copy.

There are no obvious rules for how long a chromosome segment should be (which is indicative of the TMRCA, Box 1) or for how infrequent heterozygous loci should be within chromosome segments before they are classified as being truly IBD. In practice, this problem is often dealt with by analyzing only relatively long putatively IBD segments likely to arise from recent ancestors, and excluding very short putatively IBD segments which are frequent in all individuals and generally arise from ancestors in deep history (e.g. McQuillan et al., 2008). These very short IBD segments arising from very distant ancestors are more difficult to distinguish from stretches of homozygous genotypes arising simply due to chance. Additionally, it is likely that only a tiny fraction of the variance in *F* among individuals is due to IBD segments arising from very distant ancestors. An appealing alternative solution (which requires additional work) is to define individual inbreeding parametrically in terms of the genomewide distribution of the TMRCA, an approach where it is unnecessary to categorize loci as being IBD or non‐IBD (Speed & Balding, 2015).

## 3 Estimating Individual Inbreeding (*F*)

### 3.1 The pedigree inbreeding coefficient: *F*_P_

While *F*_P_ was originally considered a parameter, in practice *F*_P_ calculated from an available pedigree is actually used as an estimator of *F* arising from ancestors included in the pedigree (Kardos, Luikart, et al., 2015; Keller et al., 2011; Wang, 2016). Useful values of *F*_P_ generally require pedigrees including several generations. Pedigrees including few generations (e.g. a three‐generation pedigree which only includes the grandparents) are likely to miss many recent ancestors that contribute to contemporary inbreeding. Behavioral and molecular genetic data collected over long periods of time can be used to construct multigeneration pedigrees (Blouin, 2003; Pemberton, 2008). In principle, it is possible to construct pedigrees in populations without long‐term behavioral data by assigning parentage based solely on analysis of molecular markers (Blouin, 2003). However, *F*_P_ estimated from marker‐based pedigrees tends to be highly downwardly biased and imprecise unless it is possible to sample all individuals in a population over many generations (Taylor, Kardos, Ramstad, & Allendorf, 2015).

### 3.2 Using unmapped molecular markers to estimate *F*

Molecular marker‐based measures of *F* range from simple estimates of individual heterozygosity to more advanced methods that use mapped loci to estimate *F* via identification of IBD chromosome segments as stretches of homozygous genotypes at mapped SNPs (i.e. “runs of homozygosity” [ROH]; Box 1). Measures of *F* based on individual heterozygosity are rooted in the idea that individuals whose parents are more closely related will have lower heterozygosity on average across the genome due to the presence of IBD chromosome segments. The simplest of the heterozygosity‐based measures of individual inbreeding is multiple‐locus heterozygosity (MLH), which is calculated as the proportion of genotyped loci that are heterozygous (Szulkin, Bierne, & David, 2010). MLH can be thought of as an estimator of the true proportion of heterozygous loci across the genome (*H*), which is a function of *F* and the heterozygosity of noninbred individuals (*H*_0_): *H *= *H*_0_(1 − *F*) (Crow & Kimura, 1970). MLH is therefore an indirect measure of *F*. Several other molecular measures of *F* were developed when most evolutionary and conservation genetics studies were based on microsatellite analysis (Amos et al., 2001; Coltman, Pilkington, Smith, & Pemberton, 1999; Coulson et al., 1998; Ritland, 1996), most of which appear to be highly correlated with MLH and provide essentially redundant information (Chapman, Nakagawa, Coltman, Slate, & Sheldon, 2009). *F* can also be measured using the diagonal elements of a genomic relatedness matrix (*F*_grm_) (Bérénos et al., 2016; Huisman et al., 2016; Powell, Visscher, & Goddard, 2010; Pryce, Haile‐Mariam, Goddard, & Hayes, 2014; Yang et al., 2010), which can be calculated with unmapped loci. Similar to other marker‐based measures of *F* (Chapman et al., 2009), *F*_grm_ tends to be highly correlated with MLH (e.g. *r *=* *.94, Huisman et al., 2016).

The usefulness of marker‐based approaches to measure *F* and to quantify inbreeding depression depends strongly on the number of loci and their expected heterozygosity, and the variance of *F* (Kardos, Luikart, et al., 2015; Miller et al., 2014; Slate et al., 2004; Szulkin et al., 2010). Until recently, MLH and similar marker‐based measures have generally had low precision because most studies have used small numbers of loci (e.g. usually a few dozen or fewer microsatellites) (Balloux, Amos, & Coulson, 2004; Taylor, 2015). Very large numbers of single nucleotide polymorphisms (SNPs) can now readily be analyzed in any organism, which provides greater precision. A major advantage of the statistics discussed above is that marker mapping information is not required, facilitating analyses in the majority of species where linkage maps are not available. A limitation of using unmapped loci is that it is impossible to explicitly identify IBD chromosome segments.

### 3.3 Using mapped loci to estimate *F*

Individual inbreeding causes discrete chromosome segments to be IBD (Figs 1 and 2, Box 1). Putatively IBD chromosome segments can be identified by detecting ROH at mapped loci (Box 1). The availability of many thousands of typed loci with known physical or genetic positions in the genome enables estimation of *F* as the proportion of the genome that is in ROH (*F*_ROH_; Curik, Ferenčaković, & Sölkner, 2014; McQuillan et al., 2008). Several approaches have been taken to identify ROH. For example, the ‐*homozyg* function in the program PLINK identifies ROH that satisfy user‐defined criteria regarding the density of SNPs, the number of allowed heterozygous positions, and minimum length (Purcell et al., 2007).

Another approach to identify ROH is to calculate the ratio of the probabilities of the genotypes within a window assuming the segment is IBD versus non‐IBD while accounting for allele frequencies, sequencing error rate, and the mutation rate (Broman & Weber, 1999; Pemberton et al., 2012; Wang, Haynes, Barany, & Ott, 2009). Sequencing errors and mutations are important to account for because they both cause heterozygous genotypes within otherwise IBD chromosome segments. Additionally, a pairwise sequentially Markovian coalescent model, a method that can infer variation in *N*_e_ over time, can be used to identify ROH as regions where the two homologous chromosome segments coalesce in a very recent ancestor (Palkopoulou et al., 2015). Individual inbreeding mainly due to recent ancestors can be measured by excluding very short ROH from estimates of *F*_ROH_. If inbreeding due to very distant ancestors is of interest, very short ROH can be included in estimates of *F*_ROH_ (Box 1) when SNPs are spaced sufficiently densely across the genome. Finally, *F* can be quantified using putative IBD segments identified with a hidden Markov model (Gazal, Sahbatou, Babron, Génin, & Leutenegger, 2014).

### 3.4 Is *F* better measured with pedigrees or molecular genetic data?

The availability of genomic resources and increasing recognition that *F*_P_ is an imperfect predictor of *F* have led to a renewed interest in determining whether *F* is better measured with pedigrees or molecular genetic data. Marker‐based measures of *F* have traditionally been perceived as being imprecise (Balloux et al., 2004; Pemberton, 2004, 2008; Santure et al., 2010; Slate et al., 2004). Most of the studies reporting poor performance of molecular measures of *F* relative to pedigrees analyzed few molecular markers compared to what is readily available today and did not account for the imprecision of pedigree analysis arising from finite pedigree depth and linkage (Balloux et al., 2004; Santure et al., 2010; Slate et al., 2004) (see Box 1). Indeed, a frequent approach to evaluate the precision of marker‐based measures of *F* was to estimate the correlation between MLH and *F*_P_ (*r*(MLH, *F*_P_)), with low values of *r*(MLH, *F*_P_) often interpreted as imprecision of MLH as a measure of individual inbreeding. However, *r*(MLH, *F*_P_) is expected to be less than the correlation between MLH and *F* (*r*(MLH, *F*)) because *F*_P_ is really only an imprecise (and downwardly biased) measure of *F*.

Genome resequencing based on high‐quality genome assemblies will make it possible to measure *F* with essentially no error because an individual can in principle be scored as heterozygous or homozygous at nearly every position in the genome. Once genome resequencing becomes commonplace in studies of natural populations, it will clearly be unnecessary to construct pedigrees in order to study inbreeding and inbreeding depression. Studies based on genome resequencing of relatively large samples of individuals in natural populations are beginning to emerge (e.g. Ellegren, 2014; Kardos, Husby, et al., 2015). However, most studies of natural populations are still based on molecular markers. Thus, an important question is whether *F* is better measured with large numbers of molecular markers or with *F*_P_.

### 3.5 Simulation‐based studies of the precision of *F*_P_ and marker‐based measures of *F*

Recent simulation‐based studies have invariably found that *F* is more precisely estimated with large numbers of molecular markers than with *F*_P_ (Kardos, Luikart, et al., 2015; Keller et al., 2011; Wang, 2016). For example, Keller et al. (2011) found that the number of homozygous rare alleles within an individual (i.e. the “homozygous mutation load”, a proxy for *F*) was more strongly correlated with *F*_ROH_ and two heterozygosity‐based measures of *F* than with *F*_P_ calculated from five‐generation pedigrees in simulations meant to represent human populations. Kardos, Luikart, et al. (2015) found that *F* was better predicted by marker‐based measures of *F* than by *F*_P_ in recently bottlenecked populations and in partially isolated small populations (*N*_e_ = 75), both in species with low and relatively high recombination rates (0.27 and 1.2 cM/Mb). For example, *F* was in some cases more strongly correlated with MLH measured with as few as 1,000 SNPs than with *F*_P_ measured with twenty‐generation pedigrees (Kardos, Luikart, et al., 2015). These results suggest that studies of inbreeding depression in natural populations should adopt marker‐based measures of inbreeding based on many thousands of loci rather than depending strictly on pedigree analysis.

### 3.6 Empirical tests for inbreeding depression: is power higher for *F*_P_ or molecular measures of *F*?

Evaluating the performance of estimators of *F* in empirical studies of natural populations requires indirect inference, because *F* is typically an unknown parameter. The expected correlation between an estimator of $F(\hat{F})$ and a fitness component (*w*) is (Szulkin et al., 2010)

$$r\left(\hat{F},w\right)=r\left(\hat{F},F\right)r\left(F,w\right).$$

This generates the prediction that fitness traits subjected to inbreeding depression should be most strongly correlated with the most precise measure of *F*. As a result, inbreeding depression will be easier to detect with the most precise measures of *F*. Therefore, one can test whether *F* is better measured with molecular genetic data or with a pedigree in a particular study using both molecular measures of *F* and *F*_P_ to test for inbreeding depression.

Several studies have compared estimates of inbreeding depression based on pedigrees and molecular genetic data. For example, Forstmeier et al. (2012) conducted such an analysis in zebra finches (*Taeniopygia guttata*) and found that fitness traits tended to be more strongly correlated with heterozygosity at 11 microsatellites and at >1,300 SNPs than with *F*_P_, particularly when excluding highly inbred individuals (*F*_P_ > 0.15) from the analysis. Huisman et al. (2016) recently found in an island population of red deer (*Cervus elaphus*) that six fitness components were significantly associated with *F*_grm_ (estimated with >37,000 SNPs), while only three fitness components were associated with *F*_P_. Pryce et al. (2014) found higher statistical support and larger estimates of the effect size of inbreeding depression on production traits in two breeds of cattle when using genomic measures of *F* (e.g. *F*_grm_ based on >43,000 SNPs) than when using *F*_P_ as a measure of *F*. Bérénos et al. (2016) found that inbreeding depression in Soay sheep was detected more frequently using genomic measures of *F* based on ~37,000 SNPs than when using *F*_P_ as a measure of *F*. However, the results of tests for inbreeding depression were qualitatively similar for analyses based on ~37,000 SNPs and *F*_P_ when the genomic data set was restricted to the individuals included in the pedigree‐based analysis (Bérénos et al., 2016). This suggests that the advantage of genomic measures of *F* over *F*_P_ was at least in part due to a larger available sample size in this case. Taken together, these studies provide compelling empirical evidence that inbreeding depression is more easily detected, and its magnitude more accurately estimated, with genomic measures of *F* than with *F*_P_. Additionally, they demonstrate that the availability of genomic data means it is now feasible to rigorously study inbreeding depression in study systems where pedigrees are not available. In many cases, analyses based on large numbers of molecular markers will provide more reliable estimates of *F* and of the fitness effects of inbreeding.

Molecular markers will not necessarily outperform *F*_P_ as a measure of *F* when relatively few loci are available. For example, Slate et al. (2004) found that several morphological traits were statistically significantly associated with *F*_P_, but not with MLH measured with 138 microsatellite loci in domestic sheep. This suggests that *F*_P_ may provide higher power to detect inbreeding depression than marker‐based measures of *F* when few molecular markers are used. The number of loci necessary for molecular measures of *F* to outperform *F*_P_ will depend on several factors including the allele frequencies, genotyping error rate, and the depth and quality of the available pedigree (Kardos, Luikart, et al., 2015; Miller et al., 2014).

### 3.7 Using genome sequencing in pedigreed populations to test the performance of *F*_P_ and marker‐based measures of *F*

Resequencing the genomes of many individuals in wild pedigreed populations will provide perhaps the best opportunity to empirically evaluate the performance of pedigrees and molecular markers in estimating *F* and detecting inbreeding depression. Genome resequencing means it is possible to measure *F* and genomewide heterozygosity extremely accurately. Subsets of SNPs identified in genome resequencing data can be used to measure *F* with various statistics (e.g. MLH and *F*_ROH_). Researchers can then determine whether actual genomewide heterozygosity (measured with the whole genome) and fitness components are most strongly associated with *F*_P_, or with marker‐based measures of *F* based on different numbers of loci sampled from throughout the genome.

### 3.8 Estimating fitness effects of inbreeding with genomic data

The recent availability of genomic data (e.g. SNP genotyping arrays, reduced representation sequencing, and whole‐genome resequencing) has dramatically improved our ability to precisely measure *F*, but this development has not changed the general approach to estimating effects of *F* on fitness components. The classical approach to estimate the strength of inbreeding depression is to perform a linear regression with a fitness component as the response, and *F*_P_ as the predictor (Keller & Waller, 2002; Morton, Crow, & Muller, 1956). The same approach is being used with genomic data, except replacing *F*_P_ with molecular measures of *F*. Large‐scale SNP data sets have frequently been used to estimate *F* and to test for effects of inbreeding on disease susceptibility in humans (Enciso‐Mora, Hosking, & Houlston, 2010; Keller et al., 2012; Kirin et al., 2010; Ku, Naidoo, Teo, & Pawitan, 2011; Lencz et al., 2007; McQuillan et al., 2008, 2012; Vine et al., 2009). However, studies of inbreeding depression based on genomic data in studies of natural, nonhuman populations are only just beginning to emerge (Bérénos et al., 2016; Hoffman et al., 2014; Huisman et al., 2016). Increasing use of high‐throughput sequencing and very large SNP genotyping platforms will almost certainly allow much more precise estimates of inbreeding effects in natural populations than ever before. This will improve our understanding and perhaps substantially change our view of the severity of inbreeding depression in the wild and its impact on population growth and viability.

Some recent studies using genomic data in wild populations (Hoffman et al., 2014; Huisman et al., 2016) suggest that inbreeding has had very strong effects on fitness‐related traits. Hoffman et al. (2014) found in a study of harbor seals (*Phoca vitulina*) that the deviance in parasite infection explained by heterozygosity increased by nearly fivefold (to 49%) when >14,000 SNPs were used compared to when only 27 microsatellite loci were analyzed. Inbreeding depression also appeared to be very strong in red deer, with lifetime breeding success (total number offspring produced over a lifetime) reduced by 72% and 95% (for females and males, respectively) among individuals with *F*_grm_ = 0.125 compared to individuals with *F*_grm_ = 0 (Huisman et al., 2016).

## 4 Identifying Populations Where Inbreeding Depression is Likely a Problem for Conservation

Identifying populations with high mean *F* is an increasingly important objective for conservation geneticists due to the rapid progression of global change and habitat fragmentation. The inability of natural selection to purge all deleterious recessive alleles means that inbreeding depression is expected in all populations where inbred individuals occur (Ballou, 1997; Bijlsma, Bundgaard, & van Putten, 1999; Boakes, Wang, & Amos, 2007; Byers & Waller, 1999; P. W. Hedrick & A. García‐Dorado, in press; Husband & Schemske, 1996; Trask et al., 2016; Willis, 1999). Some deleterious recessive alleles with large fitness effects are likely to be purged by natural selection. However, recessive or partially recessive alleles with small fitness effects (i.e. when *s*(0.5 − *h*) < 1/2*N*_e_, where *h* is the dominance coefficient, García‐Dorado, 2012) are much less likely to be purged because natural selection will be overwhelmed by genetic drift in this scenario. Identifying populations where mean *F* is high can be accomplished by detecting populations with low mean genomewide heterozygosity. This approach is possible because the major effect of individual inbreeding is increased offspring homozygosity (Box 1), which is the cause of inbreeding depression (Charlesworth & Willis, 2009).

**Figure 3:**
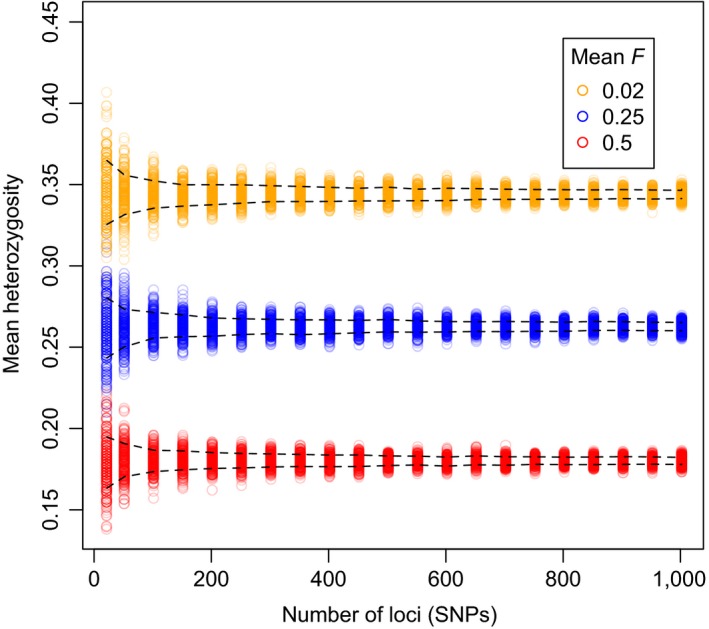
Effects of the number of loci on the precision of estimated mean heterozygosity in simulated populations. The *y*‐axis is estimated mean heterozygosity (proportion of heterozygous SNPs) in a sample of 30 individuals. The *x*‐axis is the number of SNPs used to estimate mean heterozygosity. Results from three simulated populations with different mean *F* are represented with different colors as indicated in the legend. For each population and number of SNPs, 1,000 separate nonoverlapping samples of unlinked SNPs (mean *H*_e_ = 0.3) were used to estimate mean heterozygosity among the 30 simulated individuals. The dashed lines represent the 5% and 95% quantiles of the distribution of the 1,000 estimates of mean heterozygosity. The simulations were conducted in R, using the inbreedR package (Stoffel et al., 2016). Details of the simulations are available in the Supporting Information.

Mean genomewide heterozygosity among individuals within a population can be estimated precisely by genotyping a modest number of loci spread throughout the genome on a surprisingly small number of individuals (Gorman & Renzi, 1979; Nei & Roychoudhury, 1974). For example, well under 1,000 SNPs (mean *H*_e_ = 0.3) typed in 30 individuals is sufficient to clearly differentiate populations with high versus low mean *F* (Fig. 3). Thus, it does not appear to be highly advantageous to type thousands of SNPs instead of only a few hundred loci when comparing mean heterozygosity (and thus mean *F*) across populations.

Many conservation genetics studies have compared heterozygosity among natural populations to evaluate individual inbreeding and the loss of genetic variation (e.g. Dobrynin et al., 2015; Gebremedhin et al., 2009; Li et al., 2014; Paetkau et al., 1998; Zemanová et al., 2015). A limitation of this approach is that mean heterozygosity will be reduced both in populations with recently reduced effective population size (*N*_e_) and in populations that have been small for a long time. The mean heterozygosity (and mean *F*) in a population is determined by the long‐term harmonic mean inbreeding effective population size (*N*_eI_) (Crow & Denniston, 1988). Populations that have recently become small are more likely to experience strong inbreeding depression than those that have been small for many generations (Charlesworth & Willis, 2009) because part of the genetic load is expected to be purged by natural selection over long time periods (P. W. Hedrick & A. García‐Dorado, in press). Thus, estimates of the timing and magnitude of population declines are informative and can now be obtained via analyses of large‐scale genomic data.

Several methods using genomic data are available to estimate the timing and magnitude of recent historical population declines. For example, the distribution of the lengths of ROH can be analyzed to infer population history (Kirin et al., 2010). An abundance of very long ROH suggests small *N*_e_ recently, and an abundance of very short ROH suggests small *N*_e_ in more distant history (Box 1). However, this approach (Kirin et al., 2010) is only qualitative and does not formally estimate current or historical *N*_e_. The timing of historical demographic events can be formally estimated using approximate Bayesian computation (ABC) methods (Csilléry, Blum, Gaggioti, & François, 2010), or using a diffusion approximation approach for demographic inference (Dobrynin et al., 2015; Gutenkust, Hernandez, Williamson, & Bustamante, 2009; Li et al., 2014). However, the timing and magnitude of population declines appear to be difficult to estimate using ABC analysis of thousands of SNPs (Shafer, Gattepaille, Stewart, & Wolf, 2015). The diffusion approximation approach may be inappropriate because it assumes that historical *N*_e_ was large (Gutenkust et al., 2009), which is clearly not the case for studies of strongly bottlenecked populations where inbreeding depression is a concern.

Recently, other approaches have been developed to infer recent historical fluctuations in *N*_e_ (i.e. over the past few hundred generations) using large‐scale SNP or whole‐genome resequencing data (Boitard, Rodriguez, Jay, Mona, & Austeritz, 2016; Browning & Browning, 2015; Palamara, Lencz, Darvasi, & Pe'er, 2012; Thompson, 2013). For example, Browning & Browning (2015) presented a method that uses the distribution of lengths of chromosome segments shared IBD between pairs of individuals (Browning & Browning, 2013) to estimate a time series of *N*_e_ from a generation or two before sampling to a few hundred generations back in time. Such analyses can be used to test whether populations with low heterozygosity have recently declined, or whether population bottlenecks coincide with known environmental changes or human‐caused disturbances. The ability to estimate *N*_e_ through recent history via analysis of genomic data represents a major advancement, because such information is key to understanding the timing of population declines, and the extent to which inbreeding is likely to affect fitness in contemporary populations.

The performance of these methods to estimate recent demographic history (e.g. Browning & Browning, 2015; Gutenkust et al., 2009) has, to our knowledge, not been evaluated for populations with very small *N*_e_. Thus, a large measure of caution is appropriate when using these methods to infer historical *N*_e_ in populations where inbreeding depression is a concern. It would be very helpful to evaluate the performance of diffusion approximation and IBD‐based methods to infer recent *N*_e_ of strongly bottlenecked populations using forward time simulations.

## 5 Inferring the Genetic Basis of Inbreeding Depression

A comprehensive understanding of the importance of inbreeding depression in natural populations requires knowledge of its genetic architecture, including the number of loci involved, and the contribution of deleterious alleles versus heterozygous advantage. For example, the efficacy of purging depends on the proportion of inbreeding depression that is due to deleterious recessive alleles with large effect sizes (García‐Dorado, 2012). The efficacy of purging also depends on the proportion of inbreeding depression that is caused by heterozygous advantage, in which case the genetic load cannot be purged (Charlesworth & Willis, 2009).

Loci contributing to inbreeding depression can be detected in species amenable to inbred line backcrossing experiments (e.g. fruit flies and some plants) via quantitative trait locus mapping (reviewed in Charlesworth & Willis, 2009). However, inbred line crosses are impossible in most natural populations. An additional drawback is that laboratory conditions are likely to alter the strength and genetic architecture of inbreeding depression (Meagher, Penn, & Potts, 2000; Putten, 1999) and may therefore not reflect effects of inbreeding in the wild where conditions are likely more stressful than in the laboratory. Studying the genetic architecture of inbreeding depression in the wild generally means analyzing variation among individuals within natural populations. Below we outline several useful approaches to achieve this.

### 5.1 Identifying loci responsible for inbreeding depression

Runs of homozygosity identified within individuals are highly useful for pinpointing loci responsible for inbreeding depression. The most appropriate ROH‐based method for mapping loci contributing to inbreeding depression depends on whether the trait is simple (i.e. monogenic) or complex (i.e. governed by variation at multiple loci). For example, loci responsible for simple, recessive, monogenic diseases have been mapped in humans and livestock using homozygosity mapping (Charlier et al., 2008; Kijas, 2013; Lander & Botstein, 1987; Mayer et al., 2016; Powell et al., 2010).

The key to homozygosity mapping is that all affected individuals will be homozygous for the haplotype containing the causal mutation. Candidate regions for containing the causal mutation can be identified as chromosome segments where the same haplotype is homozygous in each affected individual, and no unaffected individuals will be homozygous for the candidate disease‐ or malformation‐causing haplotype (Kijas, 2013). For example, Charlier et al. (2008) identified a 2.12‐Mb region that was homozygous for the same haplotype in 12 cattle affected by congenital muscular dystonia 1 (CMD1), whereas none of 14 individuals unaffected by CMD1 were homozygous for this haplotype. Subsequent DNA sequencing of this region revealed a missense mutation in the *ATP2A1* gene, which encodes a fast‐twitch skeletal muscle Ca^2+^ ATPase. Further analysis showed that 81 affected individuals were homozygous for the missense mutation, whereas none of 2,000 unaffected individuals were homozygous for the mutation, strongly suggesting that the missense mutation in *ATP2A1* causes CMD1. Homozygosity mapping requires very high‐density molecular markers to reliably identify ROH in the affected individuals and explicitly assumes recessive monogenic inheritance (Charlier et al., 2008; Kijas, 2013). The density of SNPs necessary to reliably identify ROH for application in homozygosity mapping will depend strongly on the length distribution of ROH, which is a function of the recombination rate and the number of generations separating the inbred individuals and the common ancestor(s) of their parents (see Box 1).

Recessively inherited diseases or malformations have been observed in small isolated populations where deleterious alleles have drifted to high frequency. An example of such a trait in an endangered species where the causal mutation has not been identified is chondrodystrophy, a lethal form of dwarfism, in the California condor (*Gymnogyps californianus*; Ralls, Ballou, Rideout, & Frankham, 2000). Pedigree analysis suggests that chondrodystrophy is likely caused by an autosomal recessive allele (Ralls et al., 2000). Trask et al. (2016) recently found that blindness in a small population of red‐billed choughs (*Pyrrhocorax pyrrhocorax*) occurred at a frequency of 0.25 in affected families, consistent with the phenotype being caused by a single deleterious recessive allele. These are two examples of traits where homozygosity mapping based on a high‐density SNP array or whole‐genome resequencing could be helpful in efforts to localize the causal mutation, facilitating genetic management via identification of carriers. Kijas (2013) provides a practical guide to performing homozygosity mapping on SNP data using the program PLINK (Purcell et al., 2007).

Homozygosity mapping is useful for locating loci responsible for simple, recessively inherited, monogenic traits. Candidate loci responsible for inbreeding depression on complex traits (i.e. traits governed by variation at multiple loci) can be identified via genomewide association (GWA) mapping of ROH with traits of interest—an approach that does not assume monogenic inheritance. This method involves identifying associations between trait values and the incidence of ROH across the genome and has been applied in studies of humans and livestock (e.g. Howard, Haile‐Mariam, Pryce, & Maltecca, 2015; Keller et al., 2012; Pryce et al., 2014).

There are multiple approaches to conducting GWA association mapping based on ROH, and the most appropriate method will depend on the study system and the trait(s) being analyzed. For example, Keller et al. (2012) used GWA mapping of ROH in an attempt to identify loci contributing to schizophrenia in humans. They first coded each 500‐kb segment in the genome according to whether it overlapped with one or more ROH. They then conducted a logistic regression for each 500‐kb window, with schizophrenia status (affected versus unaffected) as the response, and whether the window overlapped with any ROH as the predictor variable. Pryce et al. (2014) used an ROH‐based GWA association analysis on individual SNPs. Specifically, they used a linear mixed‐effect model to test for associations between milk fat and protein yield and ROH status at each SNP (i.e. whether the SNP was contained within an ROH), after controlling for SNP additive effects, age, year, parity, and permanent environmental effects. We are not aware of any study applying homozygosity mapping or GWA mapping based on ROH in a natural population to date, likely because of the high cost of sequencing and lack of linkage information for many studies of nonmodel organisms. However, costs are decreasing and technology is improving, and these approaches certainly could help to elucidate the genetic basis of inbreeding depression in wild populations.

An important caveat regarding GWA approaches is that statistical power may be very low to identify loci contributing to inbreeding depression. Even whole‐genome resequencing and/or very large sample sizes do not ensure that any of the loci contributing to phenotypic variation will be detected via traditional GWA analyses (Kardos, Husby, et al., 2015; Spencer, Su, Donnelly, & Marchini, 2009). However, loci controlling traits with very large effects on fitness have been detected via traditional GWA analyses in wild populations, including loci affecting horn size in Soay sheep (Johnston et al., 2013) and growth rate in Atlantic salmon (Barson et al., 2015), providing some hope for efforts to identify very large‐effect loci contributing to inbreeding depression in the wild. An important component of future attempts to identify loci responsible for inbreeding depression in the wild will be to evaluate the statistical power of GWA analyses based on ROH to detect large‐effect loci in small populations where sample sizes will usually be small.

The ability to identify loci contributing to inbreeding depression via GWA association mapping will clearly depend on the allele frequencies and the magnitude of fitness effects at the causal loci. Deleterious alleles are expected to be initially rare. GWA mapping based on samples of unrelated individuals is likely to have low power when the causal deleterious recessive alleles occur at low frequencies because there will be few individuals affected by the causal allele. However, deleterious recessive alleles with large effects could reach high frequencies due to strong genetic drift in populations with very small *N*_e_ (e.g. due to recent population bottlenecks and founding events involving a small number of individuals), thus increasing the power to identify deleterious recessive alleles with large effects via GWA mapping.

Examining genomewide patterns of genetic variation in progeny produced by self‐fertilization can be used to test for inbreeding depression, and to identify genomic regions likely to contain large‐effect deleterious recessive alleles. Hedrick, Hellsten, and Grattapaglia (2016) examined the distribution of heterozygosity across the genomes of 28 *Eucalyptus grandis* progeny produced by self‐fertilization of a single individual that was heterozygous at 9,590 genes. Fifty percent of progeny are expected to be heterozygous at each locus that was heterozygous in the parent. However, heterozygosity was much higher—65.5% on average—suggesting very strong selection (via poor survival) against homozygotes. They identified six regions (up to 25 Mb in length) where one of the possible homozygotes was completely missing, which suggests that the missing haplotypes contained highly deleterious recessive alleles. A similar approach could be taken on progeny from a large number of parents in natural or seminatural experiments. Such studies would be enormously helpful for quantifying the frequency of highly deleterious alleles for viability in natural populations, and the variation in the strength of inbreeding depression among the offspring of selfing parents.

### 5.2 The difficulty of distinguishing heterozygous advantage from deleterious recessive alleles at loci causing inbreeding depression

A major focus in the study of inbreeding depression in model organisms has been to determine the relative contribution of heterozygous advantage (higher fitness of individuals with a heterozygous genotype than individuals with either homozygous genotype) versus deleterious recessive alleles. The availability of large numbers of mapped genetic markers for nonmodel species will enable tests for evidence of heterozygous advantage in natural populations. However, it will be difficult to definitively exclude pseudo‐overdominance (i.e. closely linked loci with deleterious recessive alleles in repulsion) as the underlying cause of apparent signatures of heterozygous advantage (Charlesworth & Willis, 2009; Ohta, 1971). While multiple studies of model organisms have found evidence for heterozygous advantage contributing to inbreeding depression, many of these have subsequently been shown to be cases of pseudo‐overdominance rather than true heterozygous advantage after allowing recombination to break down associations between linked deleterious alleles (Charlesworth & Willis, 2009). Thus, a measure of caution will be appropriate when interpreting apparent evidence of heterozygous advantage contributing to inbreeding depression in future studies of natural populations.

### 5.3 Identifying candidate deleterious alleles could increase the power of mapping analyses

Identifying nonsynonymous mutations with predicted deleterious effects could advance our ability to pinpoint mutations causing inbreeding depression. Putatively deleterious mutations can be identified as substitutions at evolutionarily conserved sites (i.e. sites subjected to purifying selection) using algorithms implemented in software such as PROVEAN (Choi, Sims, Murphy, Miller, & Chan, 2012), SIFT (Ng & Hinikoff, 2001), and PolyPhen‐2 (Adzhubei et al., 2010). The power to pinpoint loci contributing to inbreeding depression via GWA mapping may be increased by focusing analyses on variants with predicted deleterious fitness effects, or alternatively by narrowing lists of candidate genes based on the presence of putatively highly deleterious alleles. Such analyses have frequently been applied in studies of the genetic basis of disease in humans (e.g. Domitrz, Kosiorek, Żekanowski, & Kamińska, 2016; Royer‐Bertrand et al., 2015) and are beginning to emerge in genetic studies of poor phenotypic performance in highly inbred wild populations (Dobrynin et al., 2015).

## 6 Are Pedigrees and Small Numbers of Genetic Markers Still Useful in the Study of Inbreeding Depression?

Although it is clear that *F* can be more precisely measured with molecular genomic data than with pedigrees, there are still valuable applications of pedigree information in studies of inbreeding depression. For example, the founder‐specific partial pedigree inbreeding coefficient can be used to determine whether a large fraction of inbreeding (Hedrick, Hoeck, Fleischer, Farabaugh, & Masuda, 2015) and inbreeding depression is driven by haplotypes arising from only one or a few pedigree founders (Allendorf, Hohenlohe, & Luikart, 2010; Casellas, Piedrafita, Caja, & Varona, 2009). Strong founder‐specific inbreeding depression suggests that large‐effect deleterious recessive alleles are segregating in the population (Lacy, Alaks, & Walsh, 1996). Thus, pedigree analysis is highly useful in determining whether inbreeding depression is largely due to deleterious recessive alleles with large effects. Genomic data could potentially be combined with analysis of founder‐specific pedigree inbreeding to pinpoint chromosomal regions and haplotypes harboring deleterious recessive alleles with large effects. Additionally, pedigree information will continue to be useful in testing for effects of parental inbreeding on offspring fitness (Bérénos et al., 2016).

Both *F*_P_ and small numbers of genetic markers (i.e. a few dozen microsatellites) can still be used to estimate *F* and to detect inbreeding depression. However, the results of studies of inbreeding depression based on *F*_P_ and small sets of markers should be cautiously interpreted considering that statistical power to detect inbreeding depression is likely to be low and that estimates of the effect sizes of inbreeding depression (i.e. proportion of variance in fitness explained by inbreeding) may be downwardly biased. Recent results from simulations (Kardos, Luikart, et al., 2015; Keller et al., 2011; Wang, 2016) and empirical analyses (Bérénos et al., 2016; Hoffman et al., 2014; Huisman et al., 2016; Pryce et al., 2014) clearly suggest that the best way forward for the study of inbreeding depression in the wild is the widespread use of large‐scale genomic data to measure *F*.

## 7 Conclusion

Genomic data make it possible to measure *F* with far greater precision than was previously possible with only a handful of genetic markers or even with extensive pedigrees. This advancement could fundamentally change our understanding of inbreeding depression in the wild. The availability of genomic data means it is no longer necessary to construct pedigrees to study inbreeding depression in natural populations. Dramatically increased precision of estimates of genomewide heterozygosity and *F* based on large‐scale genomic data (Kardos, Luikart, et al., 2015; Keller et al., 2011; Wang, 2016) is increasing the power to detect inbreeding depression and to reliably estimate its strength (Bérénos et al., 2016; Hoffman et al., 2014; Huisman et al., 2016; Pryce et al., 2014). A few recent studies have detected very strong inbreeding depression via analyses of genomic data (Hoffman et al., 2014; Huisman et al., 2016). If the traditional practice of using pedigrees or few genetic markers generally results in low statistical power and downwardly biased estimates of the strength of inbreeding depression, it would likely mean that inbreeding depression is stronger and more frequent in natural populations than previously thought. Future studies should further test this idea by comparing effect size estimates of inbreeding depression based on *F*_P_ and genomic measures of *F*.

A major priority for conservation biology is to more fully understand the effects of individual inbreeding on population growth and viability. Although it is clear that inbreeding depression is universal, there is still an insufficient understanding of the magnitude of the effects of inbreeding on individual fitness and, more importantly, on population growth and viability in natural populations (Allendorf et al., 2010; Johnson, Mills, Wehausen, Stephenson, & Luikart, 2011; Ouborg, 2009). Addressing this priority will require many studies with reliable estimates of fitness and *F* for many individuals. Because *F* can now be measured precisely for any individual, collecting data on enough fitness components from large numbers of individuals has become the biggest roadblock to a comprehensive understanding of the effects of inbreeding on individual fitness and population growth and viability. Thus, long‐term studies of natural populations still represent the greatest opportunity to further our understanding of the causes and consequences of inbreeding depression in the wild.

## Data Archiving

Simulation scripts for R are available in the Supporting Information.
