# Supplementary material for: Genomics advances the study of inbreeding depression in the wild
Source: Evol Appl. 2016 Oct 23;9(10):1205–18. doi: 10.1111/eva.12414 (PMC5108213; doi:10.1111/eva.12414)
Supplement: Supplementary file 1 [file EVA-9-1205-s001.docx]

**Genomics advances the study of inbreeding depressioN in the wild**

Marty Kardos, Helen R.Taylor, Hans Ellegren, Gordon Luikart, Fred W. Allendorf

**Supplementary Materials**

**Inbreeding simulations**

We used a modified version the simulation script of Kardos et al. (2015) to generate the data shown in Figures 1, 2, and 3. All simulations were done in R version 3.2.1 (R Core Team 2015).

***Simulation for Figure 1a and 1b***

For Figure 1a and 1b, we simulated the simple pedigree structure shown, with the founders (i.e. the grandparents) each having two unique copies of a single chromosome (150 Mb, 180 cM). The model follows Fisher’s theory of junctions (Fisher 1965). The number of crossovers along a chromosome during Meiosis is drawn at random from a Poisson distribution with mean and variance equal to the chromosome length (180 cM) divided by 100. The location of each crossover was determined by randomly selecting a map position along the chromosome (i.e., we assume no crossover interference). We simulated 25,000 single nucleotide polymorphisms along the chromosome (mean expected heterozygosity = 0.3), and used these to construct Figure 1b.

***Simulations for Figure* *2***

For Figure 2, we simulated three different pedigree structures designed to generate IBD chromosome segments originating from ancestors 2, 5, or 10 generations back from the end of the simulation. The pedigree structures for the simulations with common ancestors two and five generations back are shown in Figure S1 below. To simulate common ancestors 10 generations back, we extended the pedigree on the right below (common ancestor 5 generations back) to include 5 additional ancestral generations. The simulated individuals had ten 150 Mb, 180 cM chromosomes.


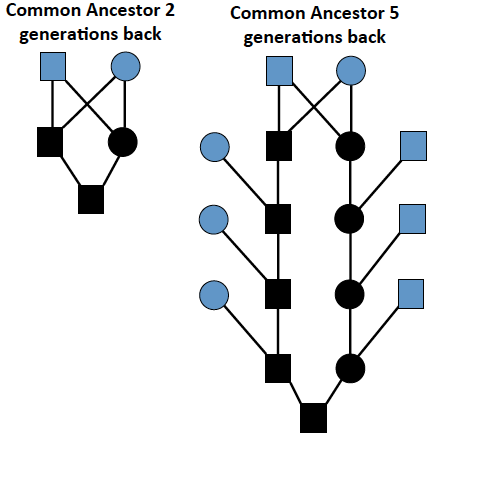


**Figure S1.** Simulated pedigree structures. The pedigree structures for simulations with common ancestors two and five generations back are shown on the left and right, respectively. Symbols filled with blue are immigrants who each had two unique copies of the simulated chromosomes. Black symbols represent residents (i.e. individuals whose parents are known). Squares represent males, and circles represent females. Sex chromosomes were not simulated. Simulations with common ancestors 10 generations back were done by increasing the number of generations of mating between the residents and immigrants. At the end of each simulation repetition, we calculated and saved the length of each IBD chromosome segment in the individual at the bottom of the pedigree. We repeated the simulations until 1,000 IBD chromosome segments were measured; the distributions of these lengths are shown in Fig. 2 in the main text of the paper.

***Simulations for Figure 3***

For Fig. 3, we simulated three populations with mean *F* among individuals of 0.02, 0.25, and 0.5. The variance of *F* was 0.01 in each of the three simulated populations. The simulated data were generated with the simulate_g2 function in inbreedR package for the program R (Stoffel et al. 2016). simulate_g2 allows the user to input the number of simulated individuals, and the mean and variance of *F* in the population the individuals are sampled from. Importantly, each estimate of mean heterozygosity in Fig. 3 is based on an independent set of loci that does not overlap with the loci included in other estimates. This was done to ensure that the sampling variance of mean heterozygosity was not underestimated due to non-independent (overlapping) sampling of loci.

**References**

Fisher, R. A. 1965. The theory of inbreeding. The theory of inbreeding.

Kardos, M., G. Luikart, and F. W. Allendorf. 2015. Measuring individual inbreeding in the age of genomics: marker-based measures are better than pedigrees. Heredity 115:63-72.

R Core Team (2015). R: A language and environment for statistical

computing. R Foundation for Statistical Computing, Vienna, Austria.

URL <http://www.R-project.org/>.

Stoffel, M. A., M. Esser, M. Kardos, E. Humble, H. Nichols, P. David, and J. Hoffman 2016. inbreedR: An R package for the analysis of inbreeding based on genetic markers. Methods in Ecology and Evolution. DOI: 10.1111/2041-201X.12588
